# Re-positioning faculty development as knowledge mobilization for health professions education

**DOI:** 10.1007/s40037-017-0362-0

**Published:** 2017-06-01

**Authors:** Stella L. Ng, Lindsay R. Baker, Karen Leslie

**Affiliations:** 1 Centre for Faculty Development, University of Toronto at St. Michael’s Hospital, Toronto, Canada; 2 The Wilson Centre, University of Toronto at University Health Network, Toronto, Canada; 3 Faculty of Medicine, University of Toronto, Toronto, Canada; 4 Li Ki Shing Knowledge Institute, Toronto, Ontario Canada; 5 Centre for Ambulatory Care Education, University of Toronto at Women’s College Hospital, Toronto, Canada

**Keywords:** Faculty development, Knowledge mobilization, Health professions education

## Abstract

Faculty development as knowledge mobilization offers a particularly fruitful and novel avenue for exploring the research-practice interface in health professions education. We use this ‘eye opener’ to build off this assertion to envision faculty development as an enterprise that provides a formal, recognized space for the sharing of research and practical knowledge among health professions educators. Faculty development’s knowledge mobilizing strategies and outcomes, which draw upon varied sources of knowledge, make it a potentially effective knowledge mobilization vehicle.

First, we explain our choice of the term knowledge mobilization over translation, in an attempt to resist the false dichotomy of ‘knowledge user’ and ‘knowledge creator’. Second, we leverage the documented strengths of faculty development against the documented critiques of knowledge mobilization in the hopes of avoiding some of the pitfalls that have befallen previous attempts at closing knowing-doing gaps.

Through faculty development, faculty are indeed educated, in the traditional sense, to acquire new knowledge and skill, but they are also socialized to go on to form the systems and structures of their workplaces, as leaders and workers. Therefore, faculty development can not only mobilize knowledge, but also create knowledge mobilizers. Achieving this vision of faculty development as knowledge mobilization requires an acceptance of multiple sources of knowledge, including practice-based knowledge, and of multiple purposes for education and faculty development, including professional socialization.

Many scholars in health professions education (HPE) have debated the relationship between education research and education practice, typically aiming to improve how education science is used in education practice [1–4]. As a field, we are not alone; countless others have examined the research-practice interface. In any arena – e. g. clinical, educational, policy – the research-practice relationship represents an important opportunity, and a continued challenge. Knowledge translation, implementation science, and knowledge mobilization are just three examples of movements aimed at closing the apparent gaps between research and practice. Knowledge translation and mobilization are generally defined as dynamic and iterative processes of synthesizing and disseminating research knowledge into practice, while implementation science refers to the study of these processes [5, 6]. Yet all of these movements continue to face questions, such as: Where does practice-based knowledge fit in, which forms of knowledge do we hope to exchange or mobilize, and who creates and uses these various forms of knowledge and to what ends? [7].

We argue that positioning faculty development *as* knowledge mobilization offers a particularly fruitful and novel avenue for the exploration of the research-practice interface in HPE. While we are not the first to make this suggestion,[8] we have yet to see it elaborated and envisioned. Therefore, we use this ‘eye opener’ to build off the suggestion[8] and envision faculty development as an enterprise that provides a formal, recognized space for the sharing of research and practical knowledge amongst health professions educators. Re-envisioning faculty development in this way both addresses and bridges the persistent research-practice gap.

To substantiate this assertion, first we explain our choice of the term knowledge *mobilization* over *translation, *in an attempt to resist the false dichotomy of ‘knowledge user’ and ‘knowledge creator’ that knowledge translation sets up. Second, we hope to avoid some of the pitfalls that have befallen previous attempts at closing knowing-doing gaps, by leveraging the documented strengths of faculty development against the documented critiques of knowledge mobilization. These arguments are meant to present a positive and novel view of the age-old research-practice gap, and to situate this view within HPE.

## Why knowledge mobilization?

We are specifically choosing the terminology of knowledge mobilization because it foregrounds a conception of fluid movement rather than hierarchical transmission of knowledge from research to practice. In making this linguistic choice, we were informed by critiques of knowledge translation that challenge its limited definition of knowledge as deriving from research alone. Such definitions are evidenced by commonly overheard conflations of ‘knowledge’ and ‘evidence’ in everyday conversation. ‘How can we move this knowledge into practice’, people ask, referring to a research-derived best practice as ‘the knowledge’.

Yet education practitioners interact with many complex social processes and systems, and thus develop and draw upon many sources of knowledge. These various conceptions of knowledge can (and should) inform practice, such as: knowledge that remains largely tacit, knowledge that derives from workplace learning, knowledge that derives from social relationships and communities, knowledge that derives from practical and ethical reasoning, and knowledge that derives from social constructions, such as learning normative ways of acting within particular cultures and contexts [9–11]. Focusing solely on ‘translating’ research knowledge into education practice fails to appreciate the realities and complexities of education practice, and the multitude of knowledges in play. This failure to fully appreciate practice-based knowledge can drive a wedge between supposed knowledge ‘users’ and ‘creators’. While *integrated* knowledge translation (IKT),[5] in the clinical world, attempted to elicit perspectives of ‘knowledge users’ at the very early stages of research development through to dissemination, it continued to suggest a knowledge hierarchy. Embedded in IKT’s terminology of knowledge *users* is an assumption that the process of knowledge translation is ultimately still about moving research knowledge, as the ‘real’ knowledge, into use in practice by practitioners as knowledge users [9]. In contrast, the language of knowledge mobilization eschews this inherent directionality and hierarchy.

This step alone – choosing knowledge mobilization as HPE’s preferred terminology – will not in and of itself instil a collaborative relationship between research and practice. So we suggest that another step toward bridging research and practice in HPE is to leverage faculty development as a form of knowledge mobilization.

## Positioning faculty development* as *knowledge mobilization

Faculty development is a form of higher education, and one well-positioned to mobilize a plurality of knowledge sources in HPE. Faculty development is often defined as a process by which faculty, including preceptors teaching in the clinical setting, work to enhance their practice in education, leadership, scholarship, personal development, and professional development. Faculty development activities should align with and support both personal *and* organizational goals,[12] since individuals do not operate in a vacuum.

While the ‘content knowledge’ being shared through faculty development programming should be up-to-date and based upon research evidence, just as importantly, faculty development programs aim to value and build upon faculty’s experiential, personal, and contextual knowledge [1, 13]. Therefore, faculty development programs often take informal, community of practice approaches, bringing together faculty through journal clubs, seminar series, and regular but informal meetings to share works-in-progress. Others offer formal or informal mentorship or a longitudinal (e. g. 2‑year) format. Research and experiential knowledge meet through dialogue between faculty developers, faculty and education researchers in these faculty development spaces. Best practices in faculty development assert that faculty bring their own expertise to the room. Faculty can then return to their local systems and influence their local HPE systems more effectively because they have discussed research-based knowledge in light of their contextualized experiences [13, 14]. Thereby, faculty development is not merely about giving individuals knowledge and skill, but also about equipping them to consider contextual and systemic factors, and socializing them to be agents of change, who can advocate for systems change.

To make our point and illustrate opportunities, we have presented faculty development as idealized, and knowledge mobilization as over-simplified. But to be fair, we recognize that there are many ready critiques of current faculty development approaches [15]. For example, one study by McLeod et al. demonstrated that regardless of having had any formal faculty development, clinical teachers demonstrated good knowledge of pedagogical principles [16]. Yet another study by the same authors showed that education scientists and clinician-educators disagreed on what they considered most *important,* pedagogically [17]. These findings in faculty development actually align with some of the fundamental critiques of traditional attempts to infuse any practice with the latest research knowledge: (1) it can be difficult to apply research knowledge directly to practice, because sound research knowledge may be unavailable for or inapplicable to some complex practice challenges, (2) contextual and systems barriers complicate individual change and (3) individuals acquire much knowledge *through *practice and informal mechanisms like their networks, but research knowledge tends not to flow through these mechanisms and networks [1, 9, 18–20]. For these reasons, faculty development should not be reduced to simplistic dissemination or provision of the best available research knowledge about education or leadership to faculty, but rather re-envisioned as a knowledge mobilizing enterprise.

Faculty development as a knowledge mobilizing enterprise re-frames this form of higher education as transformative education. In this framing, faculty development still includes the sharing of research-based and experiential knowledge on topics of interest (e. g. pedagogical or leadership practices), and also adds explicit attention to professional socialization of faculty as knowledge mobilizers and change agents [21]. By positioning faculty learners as agents of their own learning and change, faculty development can address assertions that education is insufficient to improve practices and systems [22]. We argue that health professions education (including faculty development) *narrowly defined* is indeed insufficient for large-scale change, but that education can be conceptualized as far more than providing learners with knowledge and skill [23]. Through faculty development, faculty are indeed educated, in the traditional sense, to acquire new knowledge and skill, but more importantly they are also socialized to go on to form the systems and structures of their workplaces, as leaders and workers. These empowering, transformative, and socializing processes are as much forms of education as are content knowledge and skill acquisition, and they are processes for which faculty development is well suited [24, 25]. Early research from our group suggests that faculty can indeed acquire and share new knowledge through faculty development (thus lending support for faculty development as a knowledge mobilizing vehicle). This research also shows that faculty can then go on to influence their local systems and networks (lending support for faculty themselves, as an outcome of faculty development, to become knowledge mobilizers) [14]. More explicit framing of faculty development in this image – as knowledge mobilization – requires continued, deliberate, and nuanced exploration.

## Conclusions/Next steps

Faculty development creates relational spaces for educators’ practice-based knowledge to interact with education research knowledge. Thus faculty development can be conceptualized as a form of knowledge mobilization where faculty development activities and programs bridge research and practice, and the outcomes of this bridging include faculty becoming knowledge mobilizers themselves. As knowledge mobilizers, faculty become change agents in the system. Faculty development, in this image, can build cadres of change agents who bridge research- and practice-based knowledge about education in their everyday work and leadership. We suggest that this vision of faculty development as knowledge mobilization responds to critiques of traditional knowledge translation efforts. Achieving this vision of faculty development as knowledge mobilization requires an acceptance of multiple sources of knowledge, including practice-based knowledge, in an interactional, non-hierarchical relationship. It also requires a broadened understanding of education as more than knowledge and skill acquisition; faculty development, as an educational, knowledge mobilizing enterprise, also *socializes* faculty to take on particular roles. Both faculty development and knowledge mobilization have room to grow and we suggest they may do so best, in the field of HPE, together.
